# Drug candidate identification based on gene expression of treated cells using tensor decomposition-based unsupervised feature extraction for large-scale data

**DOI:** 10.1186/s12859-018-2395-8

**Published:** 2019-02-04

**Authors:** Y-h. Taguchi

**Affiliations:** Department of Physics, Chuo University, 1-13-27 Kasuga, Bunkyo-ku, Tokyo, 112-8551 Japan

**Keywords:** Tensor decomposition, Gene expression, Feature extraction

## Abstract

**Background:**

Although in silico drug discovery is necessary for drug development, two major strategies, a structure-based and ligand-based approach, have not been completely successful. Currently, the third approach, inference of drug candidates from gene expression profiles obtained from the cells treated with the compounds under study requires the use of a training dataset. Here, the purpose was to develop a new approach that does not require any pre-existing knowledge about the drug–protein interactions, but these interactions can be inferred by means of an integrated approach using gene expression profiles obtained from the cells treated with the analysed compounds and the existing data describing gene–gene interactions.

**Results:**

In the present study, using tensor decomposition-based unsupervised feature extraction, which represents an extension of the recently proposed principal-component analysis-based feature extraction, gene sets and compounds with a significant dose-dependent activity were screened without any training datasets. Next, after these results were combined with the data showing perturbations in single-gene expression profiles, genes targeted by the analysed compounds were inferred. The set of target genes thus identified was shown to significantly overlap with known target genes of the compounds under study.

**Conclusions:**

The method is specifically designed for large-scale datasets (including hundreds of treatments with compounds), not for conventional small-scale datasets. The obtained results indicate that two compounds that have not been extensively studied, WZ-3105 and CGP-60474, represent promising drug candidates targeting multiple cancers, including melanoma, adenocarcinoma, liver carcinoma, and breast, colon, and prostate cancers, which were analysed in this in silico study.

**Electronic supplementary material:**

The online version of this article (10.1186/s12859-018-2395-8) contains supplementary material, which is available to authorized users.

## Background

Inference of compound–protein interactions is one of the important tasks of drug discovery, but the experimental approach is expensive. To slow the trend of rising drug discovery costs, computational approaches have been increasingly used. Two major in silico approaches exist: a structure-based method [1] and ligand-based one [2]. Although a lot of effort has been invested into the development and improvement of these approaches, their successes are limited, which is why alternatives are needed. One of them is the inference of target genes based on the analysis of alterations in a gene expression profile in the cells treated with the compounds of interest. Even though this approach appears to be relatively simple and straightforward, two major obstacles must be considered. It is difficult to identify drug candidate doses necessary to determine drug efficacy because there are tens of thousands of genes, and the changes in their expression must be strictly dose-dependent, otherwise any alterations are considered accidental, due to multiple-comparison adjustments. Additionally, the analysed compounds interact with proteins, and not mRNAs. Accordingly, expression of the target genes is not always affected, and therefore, gene expression profiles alone cannot provide the complete information about all the molecules targeted by the compounds under study.

To overcome these difficulties, compound signature profiling [3, 4] is often employed. In this approach, if the alterations in gene expression profiles after application of the analysed compound are similar to those observed after application of an already known drug, the compound in question is assumed to share the target genes with the previously investigated drug. With this approach – because it does not require identification of dose dependence or the target genes – the above difficulties do not apply. Nevertheless, training (labelled or annotated) gene expression datasets are required, and only previously known drug–protein interactions can be inferred.

Some examples of tasks aimed at identifying new drug–target interactions in gene expression data on the basis of known interactions are as follows. Wang et al. [5] tried to identify on- and off-target genes of drugs using similarities in drug-induced in vitro gene expression changes. Iwata et al. [6] explored potential target proteins with cell-specific transcriptional similarity using a chemical protein interactome. Lee et al. [7] tried drug repositioning for cancer therapy based on large-scale drug-induced transcriptional signatures. Although these are only a few examples, these strategies require pre-knowledge about drug–target interactions. Alternatively, instead of drug–target interactions, drug–disease interactions are studied. For instance, Cheng et al. [8] attempted to measure the connectivity between disease gene expression signatures and compound-induced gene expression profiles. Sirota et al. [9] also integrated gene expression measurements from 100 diseases and gene expression measurements for 164 drug candidates, thereby determining predicted therapeutic potentials for these drugs. Iorio et al. [10] studied compound-targeted biological pathways based upon gene expression similarities. They are unsupervised approaches to some extent, but target genes cannot be exploited.

Here, the purpose was to develop a new approach that does not require any pre-existing knowledge about the drug–protein interactions, but these interactions can be inferred by means of an integrated approach using gene expression profiles obtained from the cells treated with the analysed compounds and the existing data describing gene–gene interactions. In contrast to the studies listed above, this approach is unsupervised but can identify a drug’s target proteins. For this purpose, the recently proposed principal component analysis (PCA)-based unsupervised feature extraction (FE) [11–31] was extended through tensor decomposition (TD), and designated as TD-based unsupervised FE. The reader may wonder why decomposition was employed. This is because TD can simplify extensive information included in a massive dataset, and the derived simplified information can be used for drug–target interaction identification as follows. Because it was designed to target large-scale datasets that are formatted as a tensor and include hundreds of compounds used for treatment, the proposed method cannot be expected to show good performance when applied to a conventional small-scale dataset that includes fewer (typically a few tens of) drugs used for treatment and is formatted not as a tensor but in the conventional matrix form. Such datasets are easily associated with a fully labelled dataset because of their small size.

Before reporting the results, I would like to mention some other studies aimed at drug target identification via LINCS, which was used in this study as described later. O’Reilly et al. [32] proposed QUADrATiC, which was designed to identify a list of significant negative connections between LINCS and disease profiles. Although their method is also in some sense unsupervised, it requires additional external disease-related gene expression profiles, which are not necessary for drug target identification by TD-based unsupervised FE. Ji et al. [33] proposed integrated analysis involving LINCS and phospho-proteomics data resulting from treatments with various compounds; these data are not required by TD-based unsupervised FE either. Hsieh et al. [34] integrated LINCS with two additional databases that TD-based unsupervised FE does not need, whereas Cheng et al. [35] proposed integration of LINCS with genetic perturbations that TD-based unsupervised FE does not use either. Wolf et al. [36] employed only LINCS to identify useful drugs without target protein identification that TD-based unsupervised FE can achieve, whereas Duan et al. [37] employed LINCS and Gene Expression Omnibus, which TD-based unsupervised FE does not use. Aliper et al. [38] applied a deep neural network to LINCS with learning of external labelling that TD-based unsupervised FE does not require.

Similarly, Wang et al. [39] integrated LINCS with chemical compound structures, which are not needed for TD-based unsupervised FE. Thus, TD-based unsupervised FE appears to be the only method that can find effective drugs with identification of target proteins without any external clinical or additional expression profiles. TD-based unsupervised FE requires a list of genes affected by single-gene perturbation to identify target proteins. Because this information does not have any relation with diseases or clinical data and can be obtained by simple experimental procedures, it is the easiest information resource to get access to among those required by the other methods mentioned above.

## Results

To maximize the performance of TD-based unsupervised FE, a large-scale dataset, LINCS, which includes hundreds of drugs used for treatment of each cell line, was selected to test the performance of TD-based unsupervised FE. Although LINCS contains expression profiles for only 978 genes, the proposed strategy was designed to overcome this difficulty as well (see below).

**Fig. 1:**
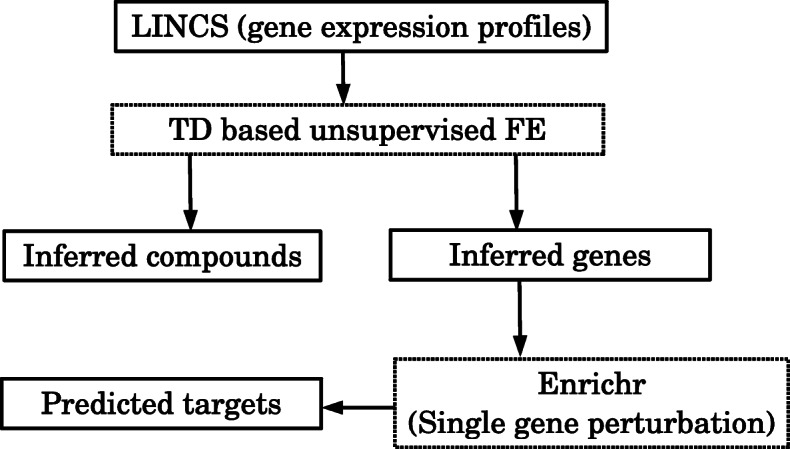
A schematic that illustrates how drug candidates and target genes are identified. Gene expression profiles retrieved from LINCS are processed by TD-based unsupervised FE. Then, ‘inferred genes’ and ‘inferred compounds’ are identified as being associated with dose dependence (this approach is detailed in Fig. 2). Then, ‘inferred genes’ are compared with a single-gene perturbation in Enrichr. Next, ‘target proteins’ are identified (this part is detailed in Fig. 3)

**Fig. 2:**
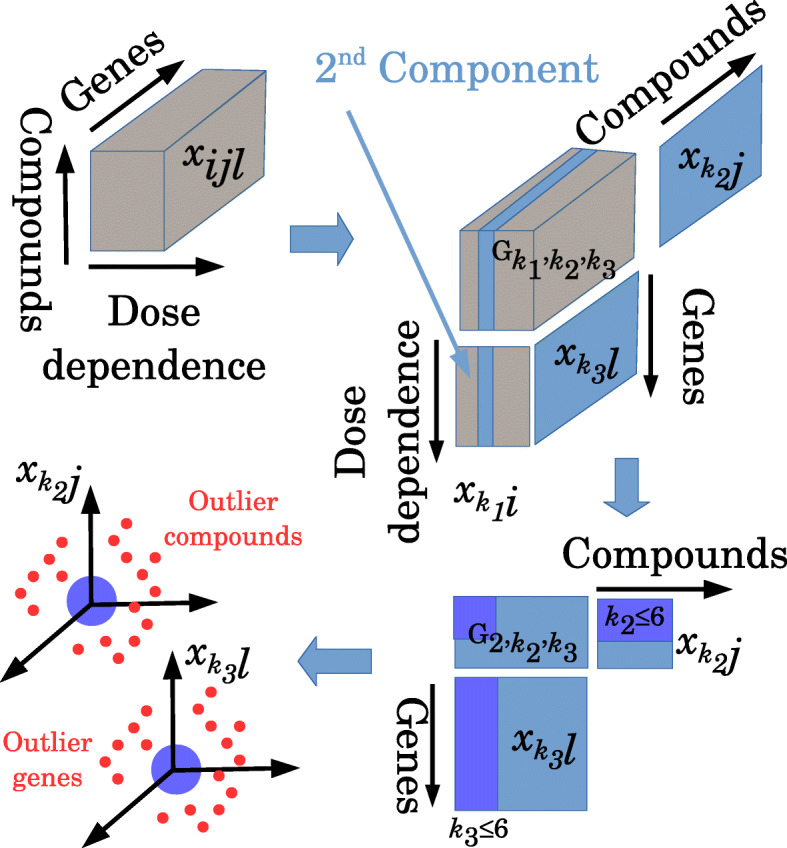
An overview of the analysis using TD-based unsupervised FE. Top left: Gene expression tensor *x*_*ijℓ*_ of dose dependence mode (*i*), compound mode (*j*), and gene mode (*ℓ*). Top right: Using the TD, *x*_*ijℓ*_, was decomposed to the tensor product of core tensor $G_{k_{1},k_{2},k_{3}}$, dose dependence matrix $x_{k_{1},i}$, compound matrix $x_{k_{2},j}$, and gene matrix $x_{k_{3},\ell }$. Bottom right: Because the second component of dose dependence mode shows linear dose dependence (Additional file 2), and cumulative contribution of the core matrix up to the sixth components exceeds 95% of the total contribution, core matrix $G_{k_{1}=2,k_{2} \le 6,k_{3} \le 6}$ is considered for FE. Bottom left: Outlier compounds (they correspond to ‘inferred compounds’ in Table 1) and outlier genes (they correspond to ‘inferred genes’ in Table 1) are identified within the space restricted with $x_{k_{2} \le 6,j}$ and $x_{k_{3} \le 6,\ell }$, respectively

Initially, the sets of genes (‘inferred genes’) showing significant dose–response relations with the compounds under study, as well as the compounds (‘inferred compounds’) showing a dose-dependent activity, were identified by TD-based unsupervised FE (this process is illustrated in Figs. 1–2, and the results are summarised in Table 1). Here, a dose-dependent activity is defined as a significant correlation observed between gene expression alterations and dose density of the compound being analysed (It can be seen as a second dose-dependent singular value vector, see Additional files 1 and 2).

**Table 1:** The number of the inferred compounds and inferred genes associated with significant dose-dependent activity

Cell lines	BT20	HS578T	MCF10A	MCF7	MDAMB231	SKBR3
Tumour	Breast					
Inferred genes	41	57	42	55	41	46
Inferred compounds	4	3	2	6	5	6
All compounds	110	106	106	108	108	106
Predicted targets	418	576	476	480	560	423
Cell lines	A549	HCC515	HA1E	HEPG2	HT29	PC3
Tumour		Lung	Kidney	Liver	Colon	Prostate
Inferred genes	45	46	48	54	50	63
Inferred compounds	8	5	7	2	2	9
All compounds	265	270	262	269	270	270
Predicted targets	428	352	423	396	358	439
Cell lines	A375					
Tumour	Melanoma					
Inferred genes	43					
Inferred compounds	6					
All compounds	269					
Predicted targets	421					

The target genes predicted by means of the comparison with the data showing upregulation of the expression of individual genes (‘predicted targets’) are also shown. The full list of inferred genes and predicted targets is available in Additional file 7. Inferred compounds are presented in Table 2. ‘All compounds’ rows represent the total number of compounds used for the treatment of each cell line

**Table 2:** Compound–gene interactions presented in Table 1 that significantly overlap with interactions described in two datasets

Compounds	(1)	(2)	(3)	(4)	(5)	(6)	(7)	(8)	(9)	(10)	(11)	(12)	(13)
Dabrafenib													○
													○
Dinaciclib							○	○	○	○	○	○	○
							○	○	○	○	○	○	○
CGP-60474			○	○	○	○	○		○			○	○
			×	×	×	×	×		×			×	○
LDN-193189	○				○								○
	○				○								○
OTSSP167							−	−		−		−	−
							○	○		○		○	○
WZ-3105		−		−	−		−	−	−			−	−
		○		○	○		○	○	○			○	○
AT-7519				○		○		○	○			○	
				○		○		○	○			○	
BMS-387032				○		○	○		○				
				○		○	○		○				
JNK-9L									○				
									○				
Alvocidib	○	○	○	○	○	○			○				
	−	−	−	−	−	−			−				
GSK-2126458							−					−	
							−					−	
NVP-BEZ235							○					○	
							×					×	
Torin-2							×					×	
							○					○	
NVP-BGT226					−			−			−	−	
					−			−			−	−	
QL-XII-47	−												
	−												
Celastrol	○												
	−												
A443654		○		○									
		○		○									
NVP-AUY922					×	○							
					−	−							
Radicicol						○							
						−							

For each compound in the table, the upper row: the drug2gene.com dataset was used for comparisons [69], the lower row: the DSigDB dataset was used for comparisons [70]. Columns represent cell lines used in the analysis: (1) BT20, (2) HS578T, (3) MCF10A, (4) MCF7, (5) MDAMB231, (6) SKBR3, (7) A549, (8) HCC515, (9) HA1E, (10) HEPG2, (11) HT29, (12) PC3, (13) A375. ○: a significant overlap between the datasets (*P*<0.05); ×: no significant overlap between the datasets; —: no data; blank: no significant dose–response relation was identified. The confusion matrix and a full list of commonly selected genes are available in Additional file 3

**Fig. 3:**
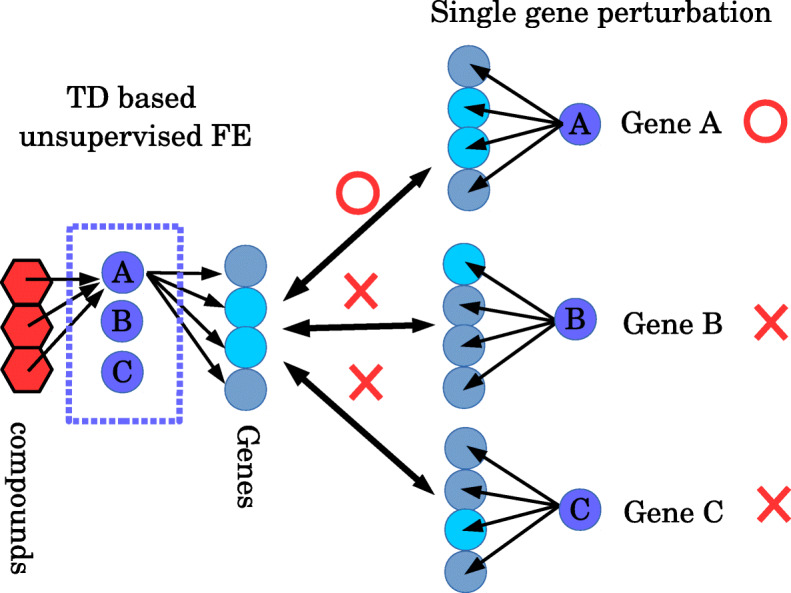
How to infer target proteins. By means of TD-based unsupervised FE, a set of genes with the expression level alterations following the activity of specific compounds can be inferred (‘inferred compounds’ and ‘inferred genes’ in Table 1), but a compound’s target genes (blue rectangle, ‘predicted targets’ in Table 1) cannot. Nonetheless, a list of inferred gene sets can be compared with that of the single-gene perturbations taken from Enrichr’s ‘Single Gene Perturbations category from GEO up’, enabling identification of the compound’s target genes

After that, to determine the genes targeted by the identified compounds (‘predicted targets’), single-gene perturbations coinciding with the alterations in gene expression profiles were identified as a consequence of the cell treatment with the analysed compounds (Fig. 3). To this end, the genes identified by TD-based unsupervised FE were uploaded to Enrichr [40] (which is the only database containing comprehensive gene expression data associated with gene knockouts [KOs]), and the genes targeted by the drug candidates were determined; these genes were afterwards assumed to be associated with the compounds targeting them.

To evaluate the predicted target genes (‘predicted target’), they were compared with two compound–target datasets, drug2gene.com and DSigDB (Table 2; the full list is presented in Additional file 3). Drug2gene.com combines the compound/drug–gene/protein information from 19 publicly available databases. DSigDB relates drugs or compounds and their target genes, for gene set enrichment analysis. As shown in the table, there is a significant overlap between the identified compound–gene interactions and the interactions present in at least one of the datasets.

One may think that significance analysis is not enough and other performance measures are beneficial, e.g. sensitivity and restricting targets to top-ranked genes. Nonetheless, this kind of analysis is not suitable for evaluation of the present results. For more details, see the discussion below.

### One hundred ninety-five genes were identified as the common compound targets, associated with dose-dependent compound activities in all the cell lines

Because 1595 unique genes are listed in Enrichr (the full list is available in Additional file 4), in the “Replacing ‘Single Gene Perturbation from GEO up’ with ‘PPI Hub Proteins’” section, the expected number of commonly selected predicted targets in all the 13 cell lines in Table 1 is at most ${1595 \times \left (\frac {600}{1595} \right)^{13} \simeq 5 \times 10^{-3}}$ (i.e. essentially zero), where 600 is the upper bound of the number of predicted targets in Table 1. Nevertheless, as shown in Additional file 4, this approach allowed for the selection of 195 common genes for further analysis.

One may argue that the assumption that 13 cell lines are independent is unrealistic because there are only seven tissues. In this case, if we assume that only seven of the 13 cell lines are independent, power should be lowered from 13 to 7. In particular, the expected number of commonly identified targets increases to as many as two. Nonetheless, because this number is still much smaller than 195, the conclusion that 195 genes should be kept for further analyses is not likely to change.

### One hundred ninety-five genes commonly identified as a compound’s target genes show enrichment with various biological terms

The information about these 195 commonly selected predicted targets was uploaded to g:Profiler [41], an additional enrichment analysis server, using 1595 genes as a background dataset. Enrichr was not used in these analyses because it cannot accept user-provided background gene datasets, and employing all the identified genes as the background is not appropriate for this method. It was found that most of the enriched gene ontology molecular function (GO MF) terms (the full list is available in Additional file 5) are related to protein–compound binding-related interactions.

### Multiple compounds bind to several proteins

Alvocidib, AT7519, BMS-387032, and dinaciclib, known cyclin-dependent kinase (CDK) inhibitors, showed a significant dose-dependent activity against the cells used in this study (Table 2). In Table 3, CDK-related proteins that are encoded by target genes presented in Table 1 are shown. Although not all CDK proteins were included in the Enrichr category, ‘Single Gene Perturbations from GEO up’, several genes encoding CDK-related proteins were identified as the targets of the compounds with the observed dose-dependent activities in all the cell lines. Recently, BRD4 was shown to bind to CDK inhibitors [42]. BRD4 and dinaciclib or alvocidib (flavopiridol) binding structures can be found in the Protein Data Bank (PDB) as PDB ID 4O71 and 4O70. In Table 3, all the cell lines where *BRD4* was found to represent a target gene (according to the results shown in Table 1) are listed. Ten out of 13 cell lines express BRD4; this result supports the finding that BRD4 binds to CDK inhibitors.

**Table 3:** Genes identified as being targeted by compounds shown to have a dose-dependent activity

Genes	(1)	(2)	(3)	(4)	(5)	(6)	(7)	(8)	(9)	(10)	(11)	(12)	(13)
CDK5RAP1					○		○				○		○
CDK9					○								○
CDK4	○	○	○	○	○	○	○	○	○	○	○	○	○
CDKN1B	○	○	○			○							○
CDK19	○	○	○	○	○	○	○	○	○			○	○
CDKN1A		○		○		○			○			○	
CDK8	○	○	○	○	○	○	○	○	○	○	○	○	
BRD4	○	○		○	○	○			○	○	○	○	○
HSP90B1												○	

Labels (1) to (13) represent the cell lines described in Table 2

To show that the obtained results – showing a good correlation between protein-binding affinity of the compounds and their activity against the cells used in this study – are not due to my preferential consideration of proteins that can bind to many compounds, radicicol was additionally analysed, a compound known to have a significant dose-dependent activity towards only one cell line, SKBR3 (Table 2). HSP90B1 was shown to bind to radicicol (binding structure: PDB ID 1U0Z), and although its dose-dependent alterations have not been observed in SKBR3 cells, they were identified in another cell line: PC3 (Table 3).

### Replacing ‘Single Gene Perturbation from GEO up’ with ‘PPI Hub Proteins’

The ‘Single Gene Perturbations from GEO up’ category can be replaced with some other criteria for further analysis. To demonstrate this strategy, the ‘Single Gene Perturbations from GEO up’ category was replaced with the ‘PPI Hub Proteins’ category in Enrichr, which shows a different interaction between genes as well. Compounds that bind to a hub protein may affect the expression of proteins that bind to that hub protein [43]. Because protein–protein interactions (PPIs) are not directly related to gene expression alterations, and the number of genes included in this category is ∼200, which is approximately 10-fold lower than the number of genes included in the ‘Single Gene Perturbations from GEO up’ category, the number of significant associations between dose-dependent activity and alterations in gene expression and compound activity obtained here was much lower (Table 4). Moreover, by means of ‘PPI Hub Proteins’, the interaction between HSP90AA1 and radicicol in SKBR3 cells was identified, which has not been observed previously because HSP90AA1 was absent in the ‘Single Gene Perturbations from GEO up’ category.

**Table 4:** A significant overlap demonstrated between compound–target interactions presented in Table 1 and drug2gene.com.

Compounds	(1)	(2)	(3)	(4)	(5)	(6)	(7)	(8)	(9)	(10)	(11)	(12)	(13)
Dinaciclib											○	○	○
CGP-60474			○	○	○	○	○		○			○	○
LDN-193189					○								
AT-7519				○		○		○	○			○	
BMS-387032				○		○	○		○				
Alvocidib		○	○	○	○	○			○				
NVP-BEZ235												○	
Celastrol	○												
A443654		○		○									
NVP-AUY922					○	○							
Radicicol						○							

In this case, the ‘PPI Hub Proteins’ category in Enrichr was used. Labels (1) to (13) represent the same cell lines as described in Table 2. The full list of confusion matrices and commonly selected genes is available in Additional file 3

## Discussion

### Identification of 195 commonly selected genes is unlikely accidental

A strong overlap that was observed between a compound’s sets of target genes identified in different cell lines supports the suitability of the proposed method. Because as many as 195 target genes (Additional file 4) were commonly identified in 13 cell lines, even though a total number of 300–600 target genes was predicted in each cell line, this result indicates that the method being tested is useful for these types of analyses.

To rule out the possibility that these overlaps were caused by non-biological factors, 50 randomly selected genes (this is a typical number of ‘inferred genes’ in Table 1) were also uploaded to Enrichr 100 times (Table 5). Considering the 100 repeats, adjusted *P*-values less than 10^−4^ were listed because 10^−4^ corresponds to 10^−2^ after adjustment for 10^2^ repetitions. At first, categories associated with adjusted *P*-vales less than 10^−4^ were detected only 20 times among 100 trials. In addition, only a limited number of categories was identified. Four times among the 20 belong to a LINCS chemical perturbation, LINCS_L1000_Chem_Pert_up/down; this outcome is in some sense inevitable because 50 randomly selected genes taken from 978 genes were tested in LINCS. Thus, identification of these categories does not have to be taken too seriously. The most frequently selected category, KEA, is kinase enrichment analysis [44]. This result is also inevitable, because 978 genes include a greater proportion of kinases than other genes. In any case, categories used to infer target proteins, ‘Single Gene Perturbations from GEO up’ or ‘PPI Hub Proteins’, were not included in Table 5. This result suggests that these overlaps are unlikely to be caused by non-biological factors that inevitably invalidate biological significance and prevent us from obtaining genes associated with significant adjusted *P*-values.

**Table 5:** Categories associated with adjusted *P*-values less than 10^−4^ among 100 trials

Enrichr Categories	Adjusted *P*-values
KEA_2013	1.56×10^−5^, 1.42×10^−5^, 1.38×10^−5^, 2.12×10^−5^
KEA_2015	1.42×10^−5^, 1.38×10^−5^
LINCS_L1000_Chem_Pert_down	9.46×10^−6^, 1.37×10^−5^
LINCS_L1000_Chem_Pert_up	3.49×10^−7^, 3.28×10^−7^
WikiPathways_2013	4.80×10^−5^
WikiPathways_2015	3.31×10^−5^, 1.30×10^−5^
WikiPathways_2016	1.30×10^−5^
GO_Biological_Process_2013	1.68×10^−5^
GO_Biological_Process_2017	5.35×10^−7^
GO_Biological_Process_2017b	5.89×10^−6^
GeneSigDB	1.16×10^−5^
BioCarta_2015	9.36×10^−6^
BioCarta_2016	9.36×10^−6^

‘Enrichr Libraries Most Popular Genes’ were selected when 50 genes randomly selected from the total of 978 genes analysed in LINCS were uploaded to Enrichr

### The identified compounds are biologically reliable

Compounds associated with a significant dose-dependent cellular response represent promising drug candidates. Such compounds are listed in Table 2, and most of the analysed compounds show an activity toward more than one type of cells. Considering that only 10 compounds or fewer per cell line were identified as active, among hundreds of tested compounds, this selection must be highly cell line-independent, and these results are unlikely to be obtained by chance; this observation corroborates the usefulness of the proposed analysis.

Detailed assessment of individual compounds identified in the present analyses further supports the usefulness of the proposed approach. The results obtained for two well-known drugs, dinaciclib and alvocidib (Table 2) were then evaluated. Dinaciclib is a well-known cyclin-dependent kinase (CDK) inhibitor, developed as a promising second-generation CDK inhibitor [45]. Alvocidib, also known by its trade name Flavopiridol, represents another CDK inhibitor and was the first such inhibitor studied in human clinical trials [46] although it is less effective than dinaciclib [45]. Alvocidib was shown to significantly affect the proliferation of primarily breast tumor cells (BT20, HST578Tm, MCF10A, MCF7, MDAMB231, and SKBR3 cell lines); this finding is somewhat expected because alvocidib was first identified as a breast cancer drug [47]. Dose–response relations were observed after application of these two drugs to seven out of 13 cell lines studied here, indicating suitability of the present analysis. On the other hand, dabrafenib was shown to have a dose-dependent activity only towards A375 cells, but, because this drug primarily targets melanoma associated with a mutation in the *BRAF* gene [48], and A375 was the only melanoma cell line included in the study, the obtained results indicate the precision of the present analyses.

### Drug candidates with a target in cancer

The usefulness of the proposed approach for identification of novel drugs was demonstrated as well, and not only for confirmation of the results obtained for previously analysed drugs. For example, the results of this study revealed that NVP-BEZ235 is active against two cell lines (Table 2). It has been shown to be a new promising drug candidate [49], and these results were obtained on H1975 cells, a non–small cell lung cancer (NSCLC) cell line. In the present study, the cells shown to be affected by NVP-BEZ235 are the A549 cell line, which is an NSCLC cell line as well, confirming the previously obtained results. AT-7519 [50], LDN193189 [51], and OTSSP167 [52] are thought to be potential new anti-cancer drug candidates. BMS-387032 has been identified as a promising drug candidate [53] as well, although its efficacy was not established in subsequent studies [54]. Because these compounds were recognized as promising in the present study as well, this observation confirms that the proposed methodology is applicable to the identification of promising drug candidates that have not been fully studied yet. Taken together, these results show that this approach can be used for confirmation of the efficacy of already studied drugs and for the identification of novel drug candidates. Based on the present analysis, CGP-60474 and WZ-3105 should be examined further as possible novel anti-cancer therapeutics. Although they were shown to significantly affect 8 cell lines here, a literature search revealed that they have not been extensively tested. Only three studies have addressed CGP-60474 [55–57], while there are no available reports about WZ-3105 efficacy.

One may wonder why these two compounds were not considered seriously. One possible reason is that they were not effective enough to treat patients as monotherapy. Actually, Wildey et al. [57] suggested that CGP-60474 should be tested with combinatorial drug therapies. Although there are no studies on WZ-3105, because our methodology fits the proposals regarding a group of compounds, these directions, i.e., combinatorial drug therapies, might be a promising strategy to make use of the drug candidates identified in the present study.

Compounds with a significant dose-dependent activity against cancer cells have considerable protein-binding affinity. Among the compounds listed in Table 2, alvocidib, AT7519, BMS-387032, and dinaciclib were analysed here as representative CDK inhibitors, and their protein-binding affinity data have been further examined [54] (see also Table 3). BRD4 was identified as a possible compound-binding protein, while HSP90B1 was shown to be able to bind to radicicol (Table 3). Therefore, it was demonstrated here that the proposed analysis can identify not only frequent compound–protein binding-related interactions but also rare interactions although perhaps not in the same cells in which the activity of the compounds was detected. Although the binding structures of many proteins and radicicol can be found in PDB (PDB ID 2Q8I: PDK3, DLAT; 2WER: HSP82; 4EGK: HSP90AA1; 2ZBK: top6A/B; 3CGY:phoQ), because they have not been included in the list of 1595 genes in the ‘Single Gene Perturbations from GEO up’ category (Additional file 4), they were not analysed further.

Taken together, the obtained findings indicate that the approach presented in this study can be used for identification of novel anti-cancer drug candidates, and for the inference of possible protein–compound binding-related interactions. One hundred and forty-six potential target genes associated with a significant dose-dependent activity in all the analysed cell lines with no known binding-related interactions with compounds were predicted here and are listed in Table 2, based on the searches performed either on drug2gene.com or in DSigDB (the full list is available in Additional file 4). Therefore, it may be worthwhile to evaluate the potential interactions between these 146 proteins and the compounds analysed in this study.

### Superiority of TD-based unsupervised FE to conventional methods

TD-based unsupervised FE is superior to conventional approaches for various reasons. Although the strategy illustrated in Fig. 2 may seem simple and efficient, to use it effectively, a researcher needs to overcome an obstacle resolved only by TD-based unsupervised FE. Namely, the set of compounds and genes associated with a significant dose-dependent activity must be identified. Because only six doses of a compound were applied, while there are millions of samples, and because there are many observed correlations between hundreds of compounds and ∼1000 genes, the obtained results showing the compound activity and the alterations of gene expression must be strictly dose-dependent. For Pearson’s correlation coefficient (PCC) calculations to be applied to determination of the significance of dose-dependent alterations, the obtained *P*-values must be as small as 1×10^−7^ for the results to remain significant even after we take into account multiple-comparison criteria. Nonetheless, this criterion corresponds to obtaining PCC as large as 0.9996, which is almost impossible. In contrast, because TD-based unsupervised FE evaluates the significance of dose-dependent activities for compounds and genes separately, the criteria for its application are not that strict and a considerable number of compounds and genes can be analysed simultaneously.

It was also shown here that different protein–compound binding-related interactions can be identified in the same cell types by applying distinct gene interaction information in combination with TD-based unsupervised FE (Tables 2 and 4). Thus, it was demonstrated that TD-based unsupervised FE can successfully identify gene–compound sets associated with significant dose-dependent activity of the compound; this task is difficult to accomplish by the existing methods.

### Overlaps between the present results and previous knowledge are significant but not very large

On the other hand, inconsistency in the prediction of compound–protein interactions between this and other studies was observed here. The results presented in the confusion matrices in Additional file 3 reveal that the consistency between previously reported compound–protein interactions and those shown in this study is not high although it is significant as shown in the above subsections. It is possible that the compound–protein interactions detected here (but not present in the analysed databases) have not been experimentally verified yet. Because there are millions of potentially active compounds, it is unlikely that the effects of all the compounds used in the present analyses of the expression of 978 genes have already been elucidated. Conversely, those interactions that are found in the datasets, but have not been detected in this study, are simply absent in either the ‘Single Gene Perturbations from GEO up’ or ‘PPI Hub Proteins’ category of Enrichr. Therefore, because additional data may be included in these categories in the future, these interactions may get validated. Hence, it is likely that a small number of common compound–protein interactions between this study and the existing data does not indicate that the proposed approach cannot be useful. To increase the consistency between these two sets of results, either the currently missing compound–protein interactions should be experimentally verified, or further information on single-gene perturbations or PPIs should be added into the databases.

This fact is also related to the reason why other performance measures like sensitivity and limiting to top-ranked genes are not suitable. Because genes not in Enrichr cannot be identified as a ‘predicted target’ in Table 1, even if all possible candidates are considered, sensitivity cannot be 1.0. In other words, sensitivity cannot be a useful measure for comparison of the performance of the other methods with the proposed method, which makes use of Enrichr. In addition, ranking is not straightforward in this study because gene KO experiments included in Enrichr have been conducted on multiple cell lines. Consequently, genes are selected if there is at least a cell line where the ‘predicted target’ is associated with an adjusted *P*-value less than 0.01. The adjusted *P*-value attributed to genes in other cell lines may be worse. This observation suggests that the adjusted *P*-value can be used to select ‘predicted targets’ but not to rank them.

### Some performance comparisons with other methods

Readers may wonder whether the performance of TD-based unsupervised FE can be compared with that of other studies if they are applied to the LINCS dataset because apparently there are many similar studies [58–60]. They may be applicable to the LINCS dataset and may overcome the above-mentioned drawback of the proposed method. Nevertheless, there are substantial differences that prevent us from directly applying these methods to the LINCS dataset. As for Noh and Gunawan’s recent study [58], using their methodology to detect a gene expression alteration caused by drug treatment, Noh and Gunawan tried to infer a transcription factor (TF) affected by drugs, by means of Enrichr, which provides the list of genes targeted by a TF. In the sense that they tried to infer a drug’s target genes by considering the coincidence between a gene expression alteration caused by drug treatment and that caused by a TF, the strategy of the above authors has some similarities with the proposed one. In contrast, because their methods require training sets that are missing for the LINCS dataset, direct application of their methodology to LINCS is impossible. Although Woo et al. [59] predict target genes by means of gene expression, they need gene-regulatory networks (GRNs) that correspond to a cell line in question. Given that there are no GRNs available for the cell lines in LINCS, Woo et al.’s methodology is not directly applicable to the LINCS dataset. Although Clark et al. [60] also predict target genes on the basis of gene expression, they need Chip data, which are absent in the LINCS dataset.

In spite of these difficulties, a question may arise whether Woo et al.’s method, DeMAND, is suitable for the LINCS dataset because they also provided as least a context-free-GRN, which can be used for any kinds of datasets, although the performance deteriorates a little if compared with a cell line-specific GRN in their study. Nonetheless, DeMAND turned out to pose additional difficulties with application to the LINCS dataset for the following reasons.

1. Although DeMAND needs multiple DMSO-treated samples that served as controls, at least triplicates and ideally six replicates, DMSO-treated cell lines included in LINCS have less than three (typically only two) DMSO-treated cell lines;
2. DeMAND cannot identify genes without gene expression profiles. This is because LINCS contains expression profiles of only 978 genes, among which genes encoding drug target proteins are rarely included;
3. DeMAND cannot specify a limited number of drugs among hundreds of drugs included in LINCS.

The methodology proposed in the present paper does not have any of these shortcomings. As for point 1, the proposed methodology does not require DMSO-treated cell lines because it attempts to identify effective drugs if there is an association with dose dependence, not via the comparison with controls. Regarding point 2, given that the proposed methodology attempts to identify drug target proteins among the genes included in Enrichr, even if they are not among the 978 genes associated with the quantified gene expression profiles in LINCS, these proteins can be identified. A possible objection is that both the proposed methodology and DeMAND were restricted because target proteins can be identified anyway by means of a prepared list (For DeMAND, 978 genes in LINCS; for the proposed methodology, genes in Enrichr). Although DeMAND requires more observations for each gene expression profile, the proposed methodology requires simple gene KO experiments that are not specific to each cell line in which gene expression profiles are analysed. As for point 3, although we can apply DeMAND only to drugs screened by the proposed methodology (i.e. ‘inferred compounds’ in Table 1), because of point 1, DeMAND cannot be applied to LINCS as is. If we use the same DMSO-treated samples as three to six replicates for DeMAND, because of point 2, this approach may identify a negligible number of target proteins (often none). In this sense, in terms of application to LINCS, the proposed methodology has obvious advantages over the existing strategies.

Because it was found that none of the existing methods can be applied to LINCS as is, it was decided to test TD-based unsupervised FE on their dataset instead of the other methods’ being applied to the LINCS dataset. Nonetheless, the proposed method, TD-based unsupervised FE, is suitable only for large-scale data where more than a hundred compounds have been tested. For the existing methods to be compared with TD-based unsupervised FE (as described below) those method will deal with a much smaller number of drugs. Thus, good performance of TD-based unsupervised FE is not expected. As for Noh and Gunawan’s study, in spite of some similarities with the present study mentioned above, there are some major differences too.

- Because Noh and Gunawan examined a connectivity map [61] (CMAP), which typically provides one dose density for each drug, tensor representation cannot be implemented.
- Given that Noh and Gunawan’s methodology cannot screen drugs, drugs of interest are pre-selected with external information (in their case, drugs whose targeted TFs are included in the database that they used, STITCH [62], were selected).

**Fig. 4:**
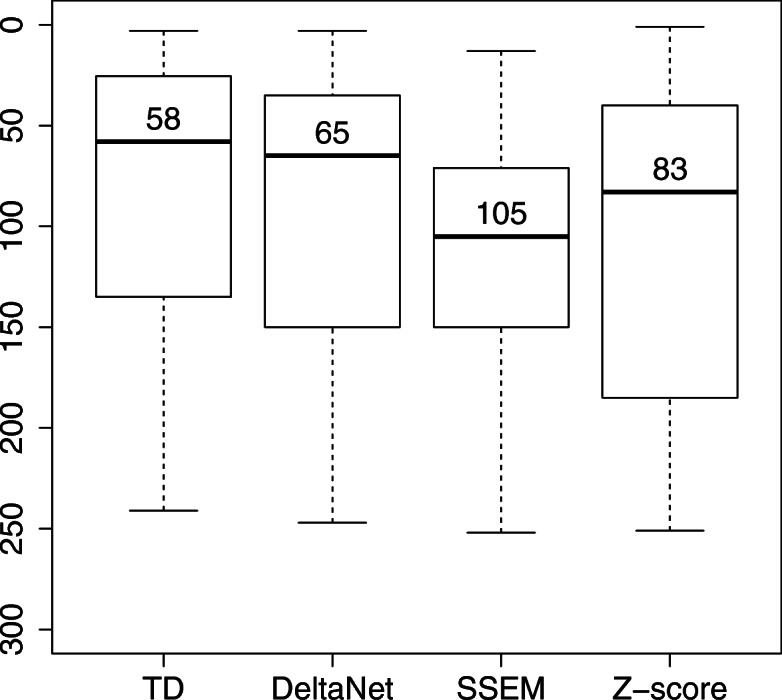
A boxplot of ranks of TFs inferred by Enrichr. The numbers are median ranks. TD: TD-based unsupervised FE, DeltaNet: Noh and Gunawan, SSEM: sparse simultaneous equation model, Z-score: Z-score–based ranking. The full list is available in Additional file 6

Thus, direct application of the present methodology, TD-based unsupervised FE, to their problem is not possible, but TD-based unsupervised FE was modified a little bit so that it is suitable to their problem. First of all, because the matrix can be regarded as a two-mode tensor, the TD-based unsupervised FE can be applicable to a CMAP dataset formally. Secondly, drug selection processes must be omitted because the drugs were pre-selected. Figure 4 shows a comparison of performance between TD-based unsupervised FE after the modification and Noh and Gunawan’s method. In brief, what they were aiming at is the following. Firstly, they tried to rank genes according to the magnitude of gene expression alteration caused by drug treatment. Then, after they uploaded top-ranked 100 genes to Enrichr, TFs were also ranked on the basis of a position weight matrix from Enrichr. Finally, the median rank of TFs that STITCH reported as targets of drugs was computed. Their procedure was repeated here using the modified proposed approach, and the outcomes were compared with theirs. As shown in Fig. 4, TD-based unsupervised FE, when applied to their problem, showed that the performance was at least comparable with that of the three methods tested by Noh and Gunawan, although superiority to the sparse simultaneous equation model (SSEM) and Z-score improved relative to DeltaNet. This is possibly because TD-based unsupervised FE was not fully adapted to the dataset analysed by Noh and Gunawan (see Additional file 6).

Applying TD-based unsupervised FE to the data of Woo et al. [59] is much simpler because their CP14 dataset is formulated as a tensor. For this trial, selection of compounds was not performed either, because Woo et al. [59] considered all 14 compounds, for which they identified 154 target genes. The results are disappointing. Although application of TD-based unsupervised FE to the CP14 dataset yielded two compounds, geldanamycin and H-7 dihydrochloride, none of the 154 target genes included in Enrichr are targeted by either of these compounds. This finding suggests that TD-based unsupervised FE is not applicable to the CP14 dataset associated with 154 target genes that Woo et al. identified. (For a more detailed description of how TD-based unsupervised FE was performed on their CP14 dataset, see Additional file 1).

These two examples where TD-based unsupervised FE was applied to a small-scale dataset – on which the existing methods have been tested – definitely show that the proposed methodology is useful only for a large-scale dataset where more than a hundred compounds have been analysed and screening of compounds is required. Thus, the existing methods pose multiple difficulties with application to LINCS as is, whereas the proposed methodology, TD-based unsupervised FE, shows some difficulties with small-scale datasets (to which the existing methods have been applied). Therefore, direct comparisons between the proposed methodology and the existing approaches proved to be problematic. It is best to regard all these methods as suitable for distinct situations; the existing approaches are suited to a small-scale dataset, whereas the proposed methodology is fine-tuned to large-scale datasets that include much greater numbers of candidate compounds. In addition, the proposed methodology does not require either control samples or comprehensive gene expression profiles, which are not available in LINCS associated with expression profiles of only 978 genes. In conclusion, in spite of the unsupervised nature, TD-based unsupervised FE is aimed at exploring a next-generation large-scale dataset like LINCS, not a classical small-scale dataset previously analysed.

### Uselessness of other more popular drug target databases for validation of targets

Readers may ask why I did not use more major drug target databases, e.g., DrugBank [63] or BindingDB [64]. The reason is simply the smaller number of target proteins included in these databases in comparison with drug2gene.com or DSigDB. Table 6 shows the list of numbers of target proteins for the compounds considered in this study. It is obvious that DrugBank and BindingDB include substantially smaller numbers of target proteins for individual drug candidates than do databases drug2gene.com and DSigDB. Because Fisher’s exact test cannot avoid yielding larger *P*-values (associated with lower statistical significance) for smaller sample sizes, there were no reasons to employ DrugBank or BindingDB instead of databases drug2gene.com and DSigDB.

**Table 6:** The numbers of target proteins of individual compounds included in four databases

Compounds	DrugBank	BindingDB	drug2gene.com	DSigDB
Dabrafenib	5	4	15	125
Dinaciclib	—	5	67	40
CGP-60474	—	8	49	16
LDN-193189	—	17	12	19
OTSSP167	—	—	—	237
WZ-3105	—	—	—	36
AT-7519	2	8	388	30
BMS-387032	—	3	392	37
JNK-9L	—	3	16	64
Alvocidib	12	31	495	—
GSK-2126458	—	5	—	—
NVP-BEZ235	—	7	76	6
Torin-2	—	10	15	15
NVP-BGT226	—	—	—	—
NVP-BGT226	—	—	—	—
Celastrol	—	6	—	89
A443654	—	3	177	104
NVP-AUY922	—	3	5	—
Radicicol	5	9	—	136

## Conclusions

The proposed method is specifically designed for large-scale datasets (including hundreds of treatments with compounds), not for conventional small-scale datasets. The obtained results indicate that two compounds that have not been extensively studied, WZ-3105 and CGP-60474, represent promising drug candidates targeting multiple cancers, including melanoma, adenocarcinoma, liver carcinoma, and breast, colon, and prostate cancers, which were analysed in this in silico study.

## Materials and Methods

### Gene expression profiles

All the gene expression profiles analysed in this study were downloaded from Gene Expression Omnibus (GEO) [65] (ID GSE70138). This super-series is composed of multiple sub-series across which a single cell line is often distributed. GSEXXXXX_series_matrix.txt.gz files included in the Series Matrix File(s) of each sub-series were downloaded; XXXXX stands for the GEO ID of each sub-series. Briefly, cell lines in which significant effects were observed 24 h after the treatment with six different doses of the investigated compounds were selected, and the maximum of 13 cell lines could be used. Gene expression levels, determined in one type of cells, after application of the same doses of compounds were averaged. If the applied-dose data were partially unavailable, these analyses were removed because TD does not permit any missing values. The numbers of compounds tested on each cell line are listed in Table 1, in the ‘all compounds’ category. Detailed information about the sub-series and cell lines used in the study is available in Additional file 1.

### TD-based unsupervised FE

In Fig. 2, an overview of the analysis is shown, and the procedure is described step-by-step in the following subsections.

#### TD

Gene expression profiles obtained for each cell line were treated as a three-mode tensor with dose-dependence mode, compound mode, and gene mode. *x*_*ijℓ*_ is the *ℓ*th expression of a gene after the treatment with the *j*th compound at the concentration of *i*. The number of different doses applied (*i*=1,…,6) and the analysed genes (*ℓ*=1,…,978) are fixed regardless of the cell line. The number of compounds used for the cell treatments varies among the cell lines, from 100 to 300 (‘all compounds’ category in Table 1). Higher-order singular value decomposition (HOSVD) was independently applied to the gene expression tensor in each cell line, and core tensors, $G_{k_{1},k_{2},k_{3}}, k_{1}=1,\ldots,6, k_{2}=1, \ldots, J, k_{3}=1,\ldots,978$, were obtained, where *J* is the number of compounds tested on each cell line, as well as three singular-value matrices corresponding to dose dependence $x_{k_{1},i}$, compounds $x_{k_{2},j}$, and genes $x_{k_{3},\ell }$, which satisfy $x_{ij\ell } = \sum _{k_{1},k_{2},k_{3}} G_{k_{1},k_{2},k_{3}} x_{k_{1},i}x_{k_{2},j}x_{k_{3},\ell }$. For more details, see Additional file 1.

#### Selection of the dose dependence mode for FE

The components coinciding with dose-dependent alterations had to be determined, to specify the dose dependence component used for FE. Here, it was observed that, regardless of the cell line analysed, the second component of the dose dependence mode always represents an almost linear dose dependence (Additional file 2). Therefore, it was decided to employ core tensors $G_{2,k_{2},k_{3}}$, as those applied to the selection of components used for FE. To identify $G_{2,k_{2},k_{3}}$ used for FE, $G_{2,k_{2},k_{3}}$ associated with exceptionally large absolute values had to be determined. To identify these $G_{2,k_{2},k_{3}}$s, independent normal distributions of $G_{2,k_{2},k_{3}}$ were assumed. Afterwards, *P*-values were attributed to all $G_{2,k_{2},k_{3}}$ values using a *χ*^2^ distribution: $ P(k_{2},k_{3}) = P_{\chi } \left [ > \left (\frac {G_{2,k_{2},k_{3}}}{\sigma _{G}} \right)^{2} \right ], $ where *σ*_*G*_ is the standard deviation of $G_{2,k_{2},k_{3}}$, and *P*_*χ*_[>*x*] is cumulative probability that the argument is greater than *x* assuming a *χ*^2^ distribution with one degree of freedom. *P*-values were then adjusted using the Benjamini–Hochberg (BH) criterion [66], which was successfully applied to *P*-values obtained by PCA-based unsupervised FE [11–31] and (*k*_2_,*k*_3_) associated with the adjusted *P*-values lower than 0.01 was selected. This approach typically resulted in ∼1,000 (*k*_2_,*k*_3_)s (the section ‘Cell lines and GEO files’ in Additional file 1). Because this number is too large to be used for FE, and the cumulative contribution of $G_{k_{1} \le 6, k_{2} \le 6,k_{3} \le 6 }= \frac {\sum _{k_{1} \le 6, k_{2} \le 6,k_{3} \le 6} \left (G_{k_{1},k_{2}, k_{3}}\right)^{2}}{\sum _{k_{1},k_{2},k_{3}} \left (G_{k_{1},k_{2}, k_{3}}\right)^{2}}, $ exceeds 0.95 for almost all cell lines, it was decided to employ (*k*_1_=2,*k*_2_≤6,*k*_3_≤6) components for FE. Nonetheless, in the case of PC3 cells, (*k*_1_=2,*k*_2_≤8,*k*_3_≤8) as an exception was applied to FE because the eighth component was found to have non-negligible contributions in this cell line.

#### FE

To identify the genes and compounds associated with a significant dose-dependent activity, it was assumed that $x_{k_{2} \le 6,j}$ and $x_{k_{3}\le 6,\ell }$ follow independent normal distributions and *P*-values were attributed to the *j*th compound and the *ℓ*th gene using a *χ*^2^ distribution, $P_{j} = P_{\chi } \left [ \!>\! \sum _{k_{2} \le 6}\! \left (\!\frac {x_{k_{2},j}}{\sigma _{k_{2}}} \right)^{2}\!\right ]$ and$P_{\ell } = P_{\chi } \left [\! > \sum _{k_{3} \le 6} \left (\!\frac {x_{k_{3},\ell }}{\sigma _{k_{3}}} \right)^{2}\!\right ]$ where $\sigma _{k_{2}}$ and $\sigma _{k_{3}}$ are standard deviations of $x_{k_{2},j}$ and $x_{k_{3},l}$, respectively. For PC3 cells, *k*_2_≤8 and *k*_3_≤8 were used in the above equation. *P*_*χ*_[>*x*] is the cumulative probability that the argument is greater than *x* assuming a *χ*^2^ distribution with eight degrees of freedom for PC3 cell lines and with six degrees of freedom for other cell lines. *P*_*j*_ and *P*_*ℓ*_ were adjusted by means of the BH criterion, and compounds and genes associated with the adjusted *P*-value lower than 0.01 were selected as those associated with a significant dose-dependent cellular response. The obtained results are listed as ‘inferred genes’ and ‘inferred compounds’ in Table 1.

### Conversion of prob IDs to the gene symbols

Because genes are identified using prob ID in a gene expression profile, whereas Enrichr accepts only gene symbols, prob IDs were converted to gene symbols using a gene ID conversion tool in DAVID [67, 68]. The conversion table is presented in Additional file 7.

### The analysis of genes obtained using TD-based unsupervised FE

As illustrated in Fig. 3, in addition to the list of genes obtained by TD-based unsupervised FE, a list of genes for identification of the association between genes showing dose-dependent alterations in the expression (‘inferred genes’) and genes targeted by the compounds shown to have a dose-dependent activity (‘predicted targets’) was required as well. Therefore, genes selected by TD-based unsupervised FE were uploaded to Enrichr, and downloaded the list of genes found in the ‘Single Gene Perturbations from GEO up’ category. The genes showing adjusted *P*-values lower than 0.01 were identified as the target genes of the analysed compounds (Table 1; ‘predicted targets’). The ‘Single Gene Perturbations from GEO up’ category was replaced with the ‘PPI Hub Proteins’ category to obtain the results presented in Table 4, by means of the same protocols.

### Previously identified compound–protein interactions

Two resources were selected: drug2gene.com [69] and DSigDB [70]. On the drug2gene.com website, if no analysed compounds could be found, then InChiKey [71] was used. Genes for which a ‘no binding’ response was obtained were excluded. Often, more than one data source came up when DSigDB was used. In these cases, data taken from LINCS [72] were generally used. For AT-7519 and BMS-387032, KINOMEscan data were employed because DSigDB does not include data from LINCS for these compounds. Only D2 (kinase inhibitors) were used.

### Evaluation of the significance of coincidence

To evaluate statistical significance of the coincidence of the interactions identified here and those previously reported, Fisher’s exact test was carried out. The number of background genes is required, but ‘Single Gene Perturbations from GEO up’ and ‘PPI Hub Proteins’ do not include all genes, and not all genes are reported in other studies. Nonetheless, it was assumed that the number of background genes was 20,000, which is considered an approximate number of human genes [73]. Data with *P*-values less than 0.05 were considered significant. Contingency tables are available in Additional file 3.

### Retrieving adjusted *P*-values attributed to ‘predicted targets’ by Enrichr

A set of ‘inferred genes’ as gene symbols was uploaded to Enrichr. Then, ‘Single Gene Perturbations category from GEO up’ or ‘PPI Hub Proteins’ were referenced, and the resulting table is downloaded. Then, genes associated with adjusted *P*-values less than 0.01 in at least one of included cell lines were identified as ‘predicted targets’.

### Statistical analysis

All the calculations were performed in the R software (version 3.3.0) [74]. Gene expression profiles downloaded from GEO were loaded into R using the read.table function. HOSVD analyses were conducted by means of the hosvd function in the R package rTensor. *P*-values were computed using the pchisq function and adjusted by the p.adjust function with the ‘BH’ option. Fisher’s test was carried out by means of the fisher.test function.

## Additional files

Additional file 1 Supporting Information with the Fig. S1 legend. (PDF 184 kb)

Additional file 2 Fig. S1 Second dose-dependent singular value vectors. (PDF 19 kb)

Additional file 3 The detailed full list of the data in Table 2. (XLSX 22 kb)

Additional file 4 1595 unique genes are listed in Enrichr. (XLSX 31 kb)

Additional file 5 Details of the GO term enrichment list. (XLSX 132 kb)

Additional file 6 A comparison with the dataset analysed by Noh and Gunawan. (XLSX 9 kb)

Additional file 7 Conversion of prob IDs to gene symbols. (XLSX 45 kb)

## Abbreviations

BH: Benjamini–hochberg
CMAP: Connectivity map
DeMAND: Detecting mechanism of action by network dysregulation
DMSO: Dimethyl sulfoxide
DSigDB: Drug signatures dataBase
FE: Feature extraction
GEO: Gene expression omnibus
GO: Gene ontology
GRN: Gene regulatory network
HOSVD: Higher-order singular value decomposition
KO: Knockout
LINCS: Library of integrated network-based cellular signatures
MF: Molecular function
NSCLC: Non–small cell lung cancer
PCA: Principal component analysis
PCC: Pearson correlation coefficient
PDB: Protein data base
PPI: Protein–protein interaction
QUADrATiC: QUB accelerated drug and transcriptomic connectivity
SSEM: Sparse simultaneous equation model
STITCH: Search tool for interactions of chemicals
TD: Tensor decomposition
TF: Transcription factor
