# Supplementary material for: Drug candidate identification based on gene expression of treated cells using tensor decomposition-based unsupervised feature extraction for large-scale data
Source: BMC Bioinformatics. 2019 Feb 4;19(Suppl 13):388. doi: 10.1186/s12859-018-2395-8 (PMC7394334; doi:10.1186/s12859-018-2395-8)
Supplement: Supplementary file 1 — Supporting Information with the Fig. S1 legend. (PDF 184 kb) [file 12859_2018_2395_MOESM1_ESM.pdf]

Supporting Information

Drug candidate identification based on gene expression of treated cells using tensor decomposition-based unsupervised feature extraction for large-scale data

Y-h. Taguchi

The number of G_{2,k_2,k_3} associated with adjusted P-values less than 0.01 in each cell line

(1)	A375	1018
(2)	HA1E	1041
(3)	A549	1335
(4)	HCC515	1204
(5)	HEPG2	1147
(6)	BT20	554
(7)	HS578T	485
(8)	HT29	1021
(9)	MCF10A	358
(10)	MCF7	496
(11)	MDAMB231	502
(12)	SKBR3	400
(13)	PC3	1390

Cell lines and GEO files

	cell lines	GEO
(1)	A375	GSE70564
(2)	HA1E	GSE70566
(3)	A549	GSE70564,GSE70565
(4)	HCC515	GSE70566,GSE70567
(59	HEPG2	GSE70567
(6)	BT20	GSE70565
(7)	HS578T	GSE70567,GSE70568
(8)	HT29	GSE70568
(9)	MCF10A	GSE70569
(10)	MCF7	GSE70569,GSE70570
(11)	MDAMB231	GSE70570
(12)	SKBR3	GSE70571
(13)	PC3	GSE70570,GSE70571

Algorithm 1 HOSVD

Require: $\sum_{i_m=1}^{N_m} A^{(m)}_{k_m,i_m} A^{(m)}_{k'_m,i_m} = \delta_{k_m,k'_m}$

Ensure: $x_{i_1 i_2,\ldots,i_n} = \sum_{\{k_m\}} G_{k_1,k_2,\ldots,k_n} \prod_{m=1}^{n} A^{(m)}_{k_m,i_m}$

$A^{(m)}_{k_m,i_m} = \delta_{k_m,i_m}$

while $\left\| x_{i_1 i_2,\ldots,i_n} = \sum_{\{k_m\}} G_{k_1,k_2,\ldots,k_n} \prod_{m=1}^{n} A^{(m)}_{k_m,i_m} \right\| > \epsilon$ **do**

for $m = 1$ to n **do**

$y_{i_m,\ell_m=\{k_{m'}\neq m\}} = \sum_{\{i_{m'}\neq m\}} x_{i_1,i_2,\ldots,i_n} \prod_{m'\neq m} A^{(m')}_{k_{m'},i_{m'}}$

$A^{(m)}_{k_m,i_m} y_{i_m,\ell_m}$: left handed singular value matrix of y_{i_m,ℓ_m}

end for

$G_{k_1,k_2,\ldots,k_n} = \sum_{\{i_m\}} x_{i_1,i_2,\ldots,i_n} \prod_{m=1}^{n} A^{(m)}_{k_m,i_m}$

end while

Mathematical details of HOSVD

Since G_{k_1,k_2,k_3} is as large as $x_{ij\ell}$, one may think that HOSVD is meaningless if we aim to reduce the degrees of freedom. In order to achieve this purpose, core tensor must have limited number of elements with large absolute values. In order that, HOSVD employs the algorithm 1.

In this algorithm, tensor is unfolded to be a matrix, y_{i_m,ℓ_m}, and singular value decomposition is applied iteratively. This supports that core tensor has limited number of elements with larger absolute values for smaller k_is.

Modification of TD based unsupervised FE so as to be applied to Noh and Gunawan's study

CMAP gene expression profiles were downloaded from the site[1]. All of CEL files are extracted. Then, each of CEL files was processed via `mas5` function in `affy` package in R. List of files[2] was also obtained. Since three platforms are used, commonly included probes were considered. Then, finally, a matrix of 22268 probes times 3073 samples for MCF7 cell lines was generated. PCA was applied to the matrix such that PC score, x_{ki}, and loading, x_{kj}, were assigned

[1]https://portals.broadinstitute.org/cmap/
[2]https://portals.broadinstitute.org/cmap/cmap_instances_02.xls

to ith probes and jth samples, respectively. In usual usage of TD based unsupervised FE, drug singular value vectors are first selected and drugs are selected using these singular value vectors. However, in Noh and Gunawan's study, drugs were already selected. Thus, drug singular value vectors, x_{kj}, associated with drugs are selected as follows. P-values are attributed to k of x_{kj} as

$$P_k = P_{\chi^2}\left[> \sum_j \left(\frac{x_{kj}}{\sigma_j}\right)^2\right]$$

where summation was taken over all of replicates, j, of the specified drug treatment. Next, gene singular value vectors, x_{ki}, must be selected in order to select genes. There are three ways for this procedure.

1. If there are ks associated with adjusted P-values less than 0.01, these ks are selected.

2. Otherwise, ks with adjusted P-values less than 0.05 were selected.

3. If the above two do not stand, five ks with smaller P_ks were selected.

Although these above is heuristic, it is inevitable so as to be applied to Noh and Gunawan's study. Finally, top ranked 100 genes associated with smaller P_i as

$$P_i = P_{\chi^2}\left[> \sum_k \left(\frac{x_{ki}}{\sigma_k}\right)^2\right]$$

were selected where summation was taken over selected ks in the above. 100 genes are uploaded to Enrichr and P-values computed by the category of "TRANSFAC and JASPAR PWMs" was used for ranking TFs.

Application of TD based unsupervised FE to CP14 data set

Gene expression profiles were downloaded from GEO (GEO ID GSE51068). Downloaded gene expression profiles were formated as tensor, $x_{ij\ell mn}$, which is the nth gene (probe) expression ($n = 1, \ldots, 49293$ after control probes with id "AFFX" were excluded), jth replicates ($j = 1, 2, 3$) at ℓth dose density ($\ell = 1, 2$) at mth time points

($m = 1, 2, 3$) of ith compounds ($i = 1, \ldots, 15$, which is 14 compounds and DSMO). HOSVD was applied to $x_{ij\ell mn}$ and compound singular value vector, $x_{k_1 i}$, replicates singular value vectors, $x_{k_2 j}$, dose density singular value vectors, $x_{k_3 \ell}$, time point singular value vectors, $x_{k_4 m}$, and gene singular value vectors $x_{k_5 n}$ were obtained. Since $x_{k_2=1,j}$ represents constant weights for all replicates, $k_2 = 1$ was selected. Since $x_{k_2=2,\ell}$ represents distinction between two dose densities, $k_2 = 2$ was selected, Since $x_{k_3=3,\ell}$ represents the monotonic time dependence, $k_3 = 3$ was selected. It was found that top ranked compounds singular vectors, $x_{k_1 i}$s, associated with larger absolute $G(k_1, 1, 2, 3, k_5)$s have larger absolute values for $i = 8$ and 14, which correspond to Geldanamycin and H-7+Dihydrochloride, respectively. Thus, these two compounds were identified as effective compounds among 14 compounds tested by TD based unsupervised FE. Nevertheless, as denoted in main text, no target proteins identified by Woo et al for these two compounds are in Enrichr's "Single gene perturbation GEO up" category. Then, no further analyses were allowed to perform.

SI Figure legends

Figure S1

Dose dependence singular value vectors, $x_{k_1,i}, k_1 = 1, 2, 3$ for 13 cell lines in LINCS. The first to the third columns are $k_1 = 1, 2, 3$. The first to the 13th rows are 13 cell lines.
