# Supplementary figures and images for: Drug candidate identification based on gene expression of treated cells using tensor decomposition-based unsupervised feature extraction for large-scale data
Source: BMC Bioinformatics. 2019 Feb 4;19(Suppl 13):388. doi: 10.1186/s12859-018-2395-8 (PMC7394334; doi:10.1186/s12859-018-2395-8)

A375

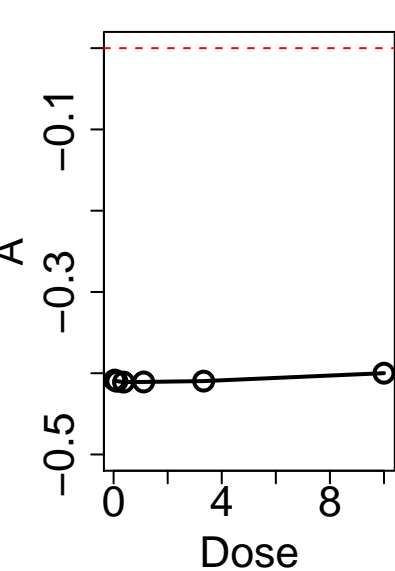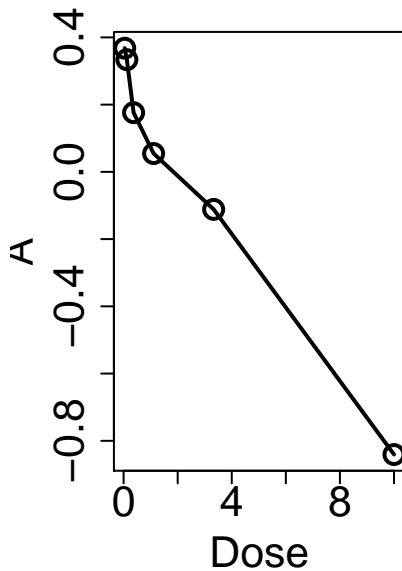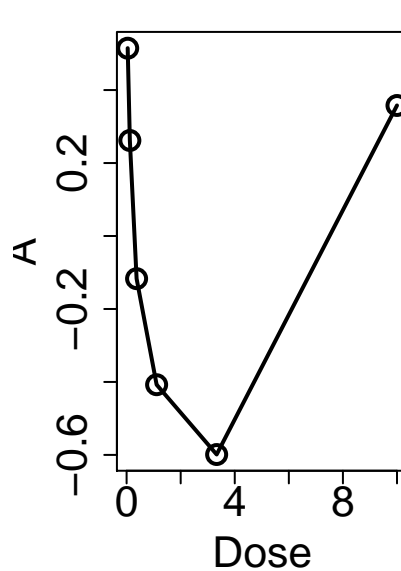

HA1E

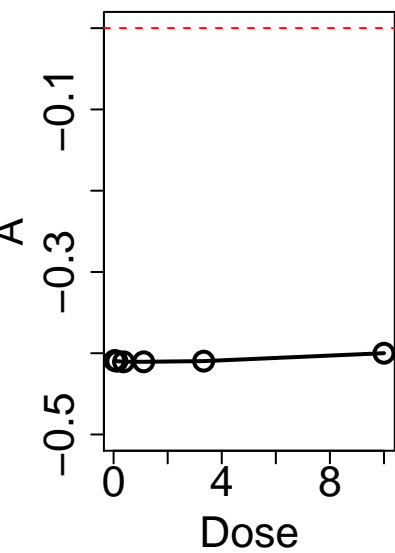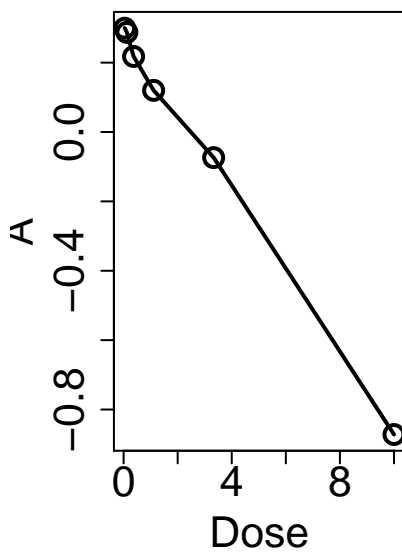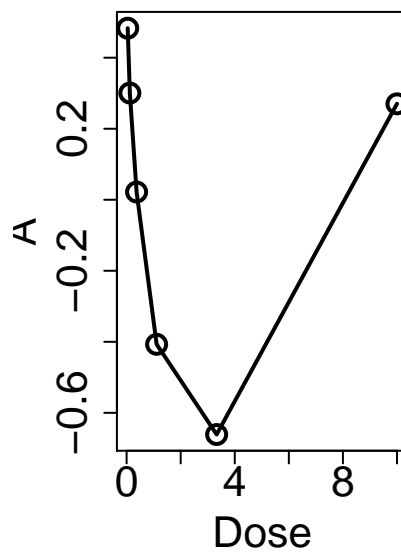

A549

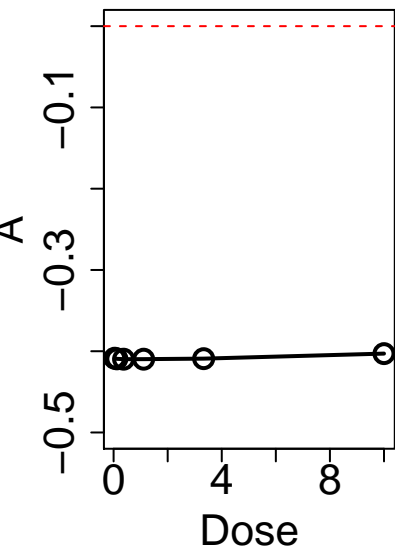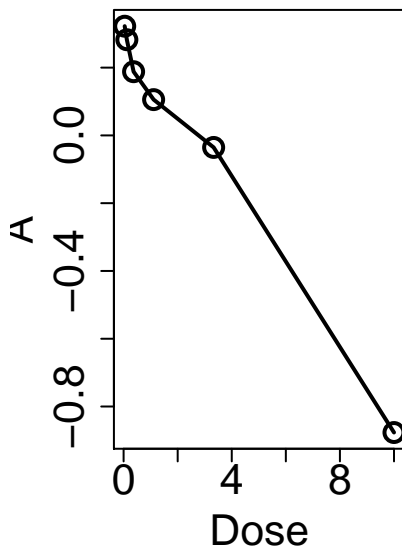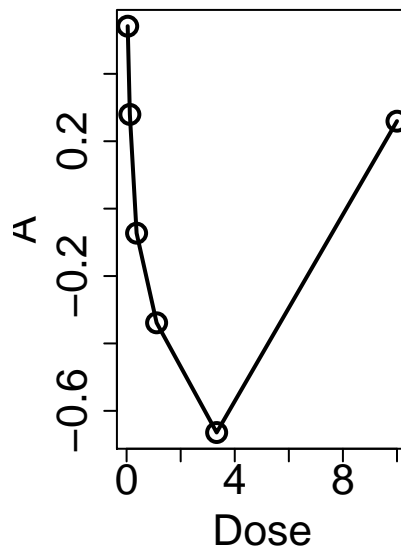

HCC515

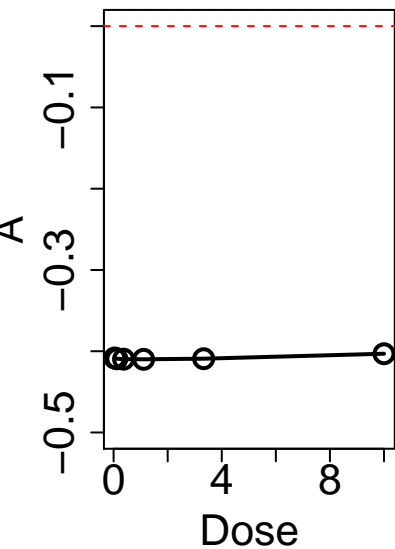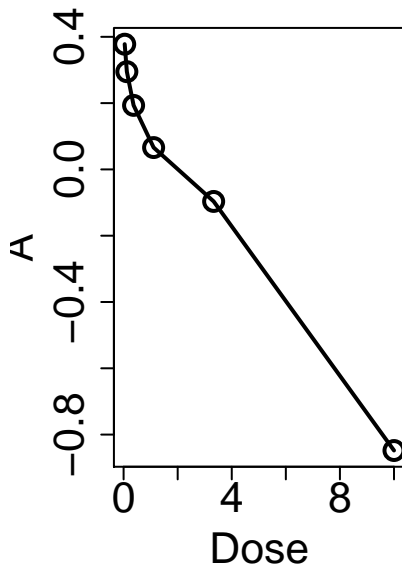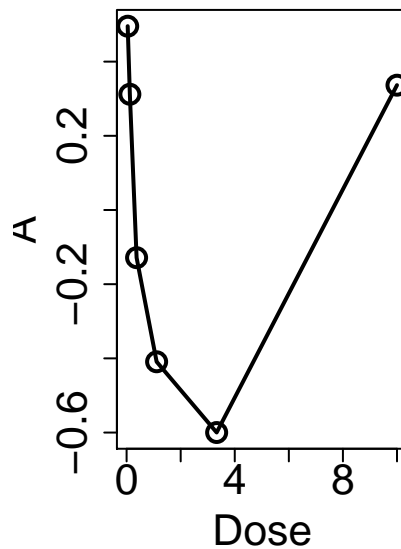

HEPG2

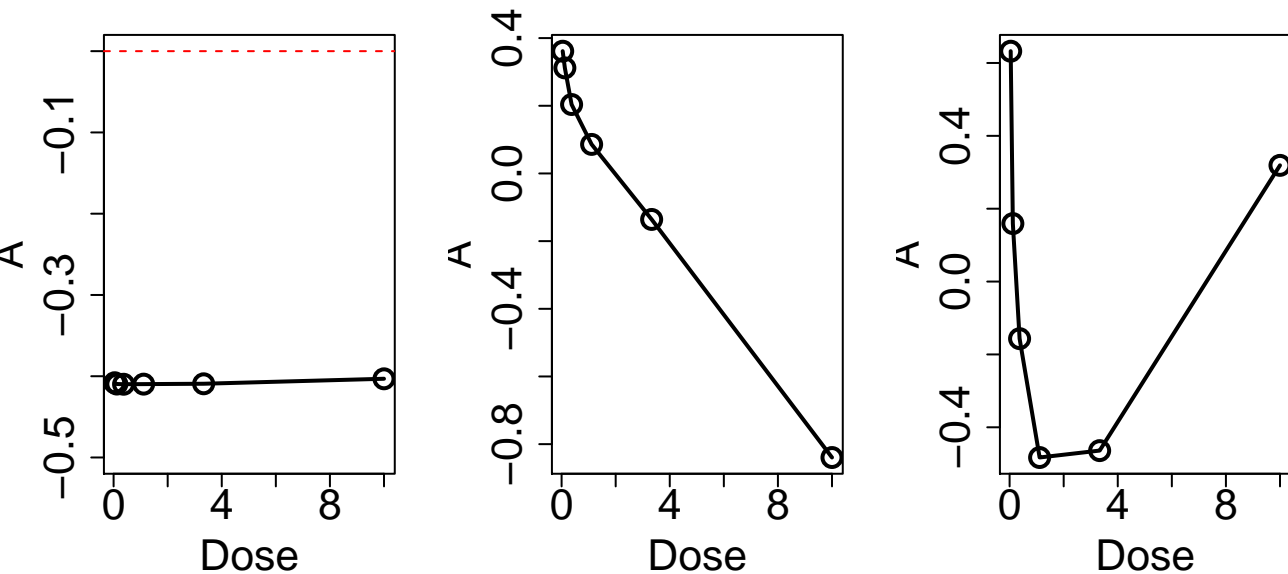

BT20

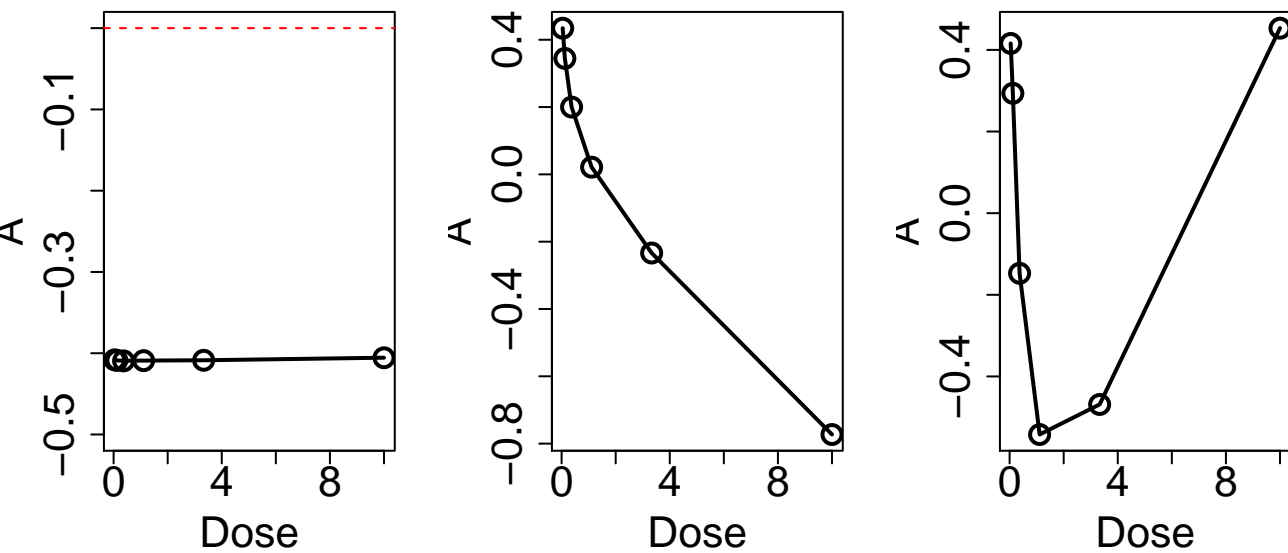

HS578T

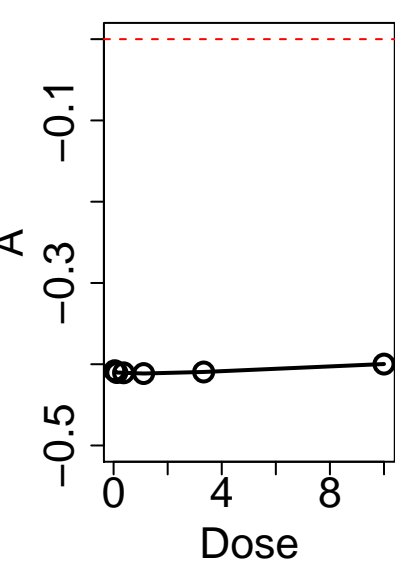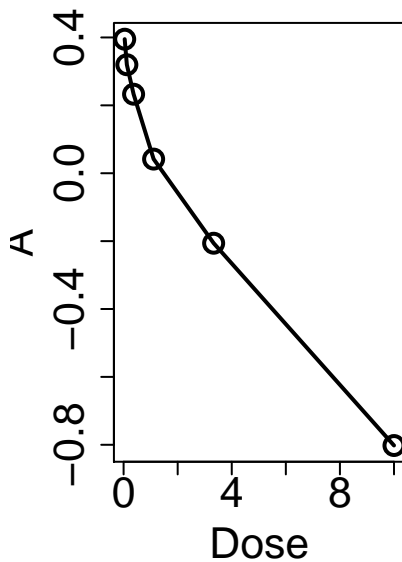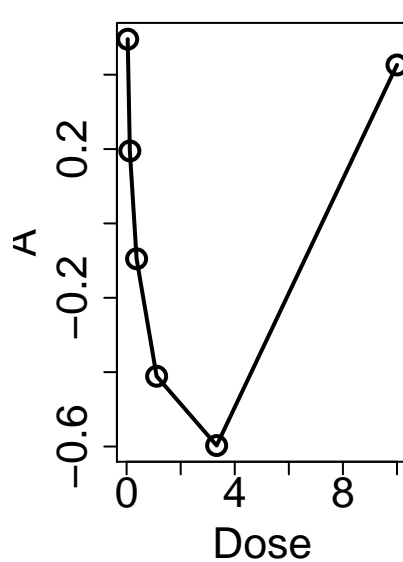

HT29

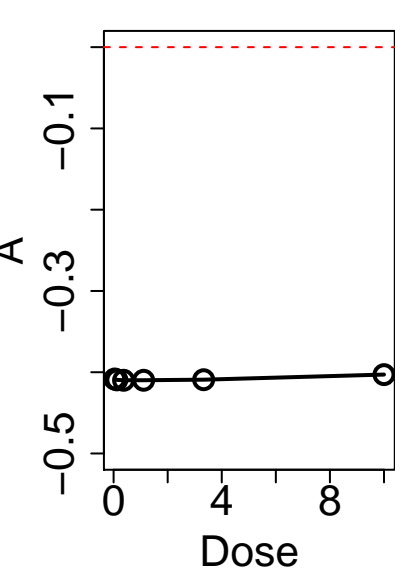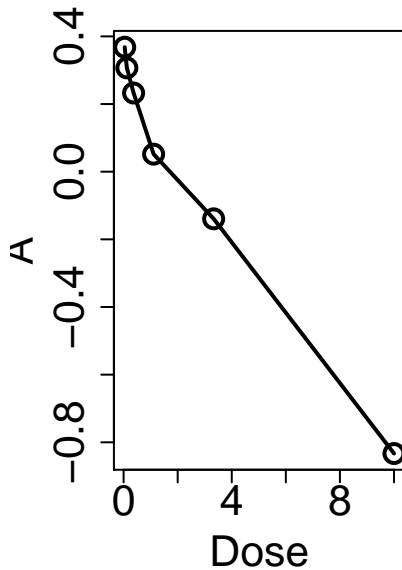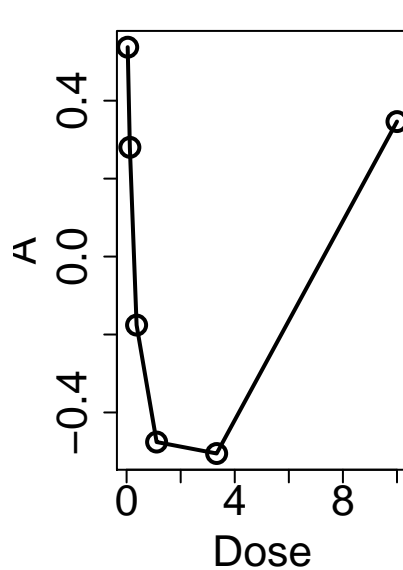

MCF10A

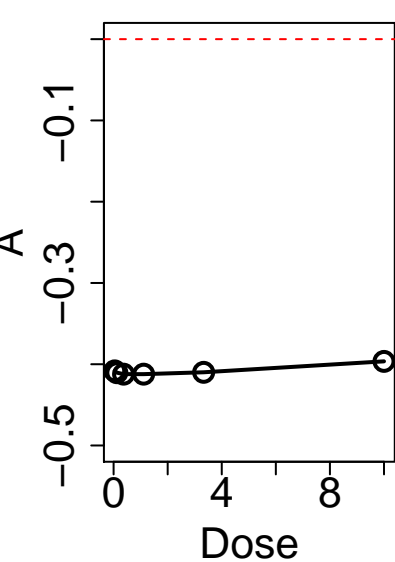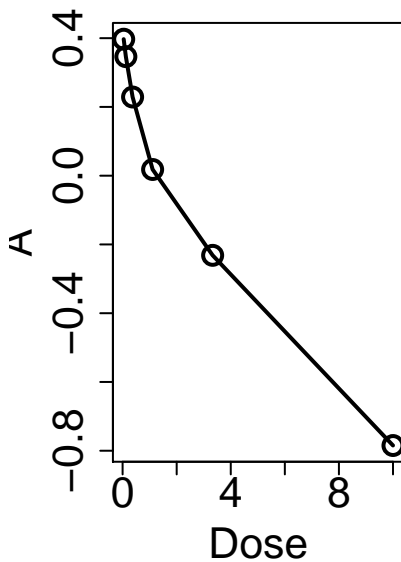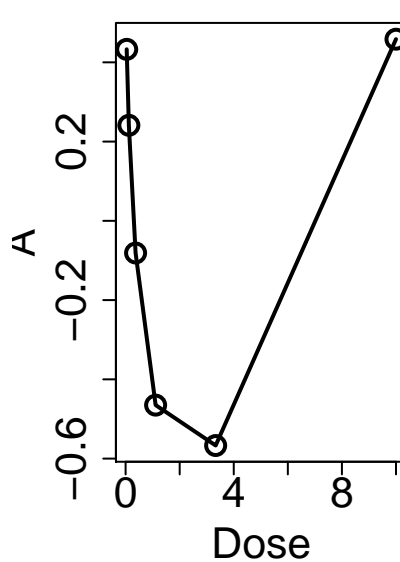

MCF7

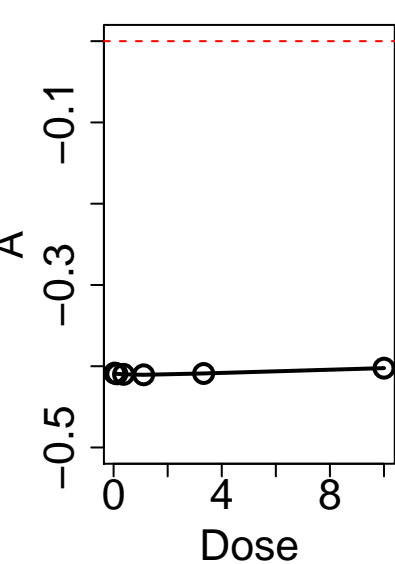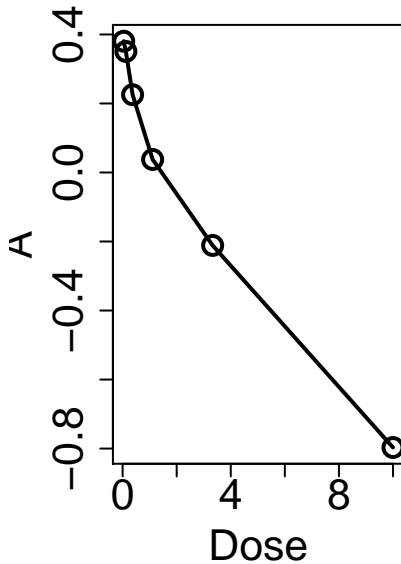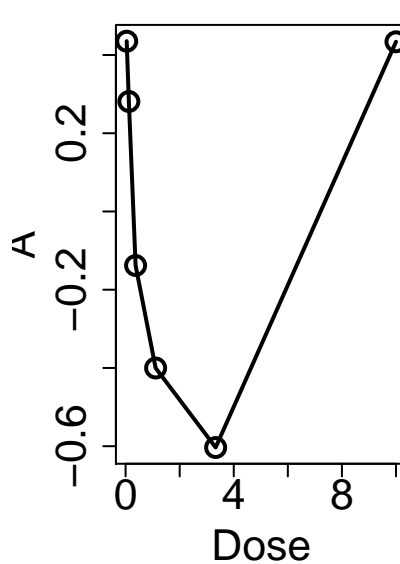

**MDAMB231**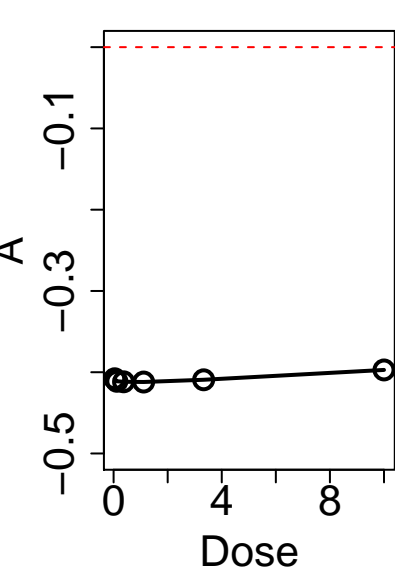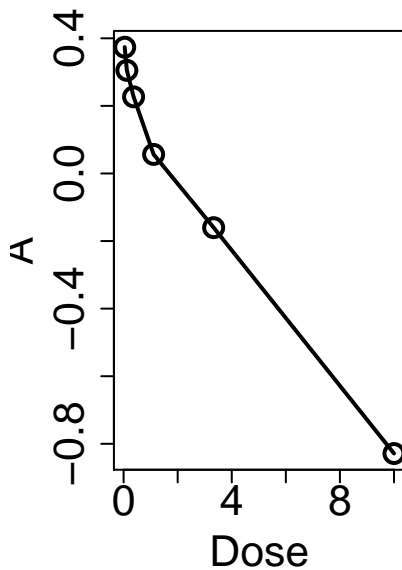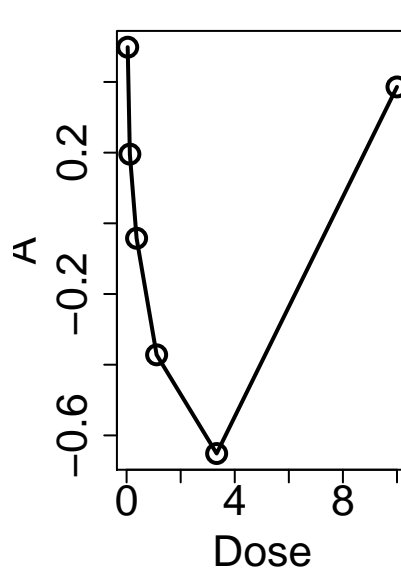**SKBR3**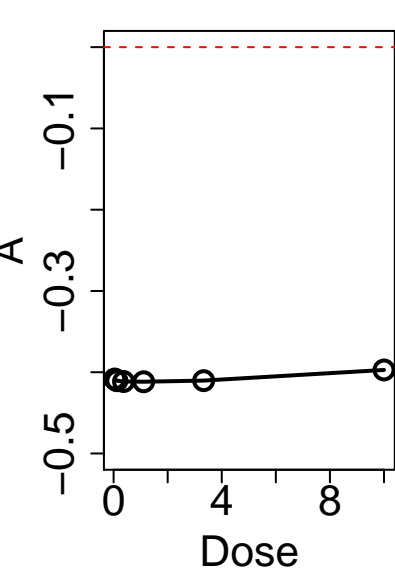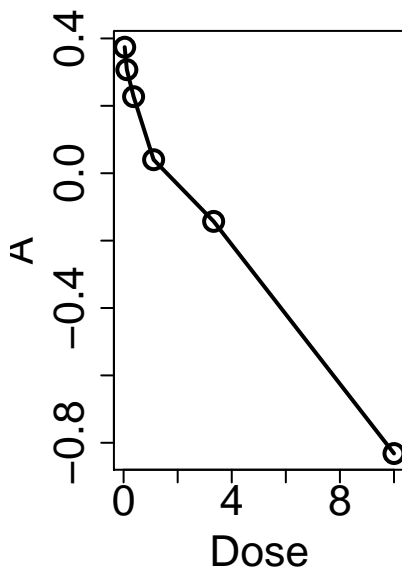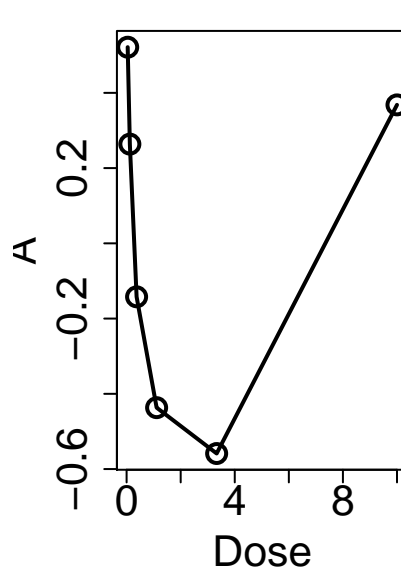

PC3

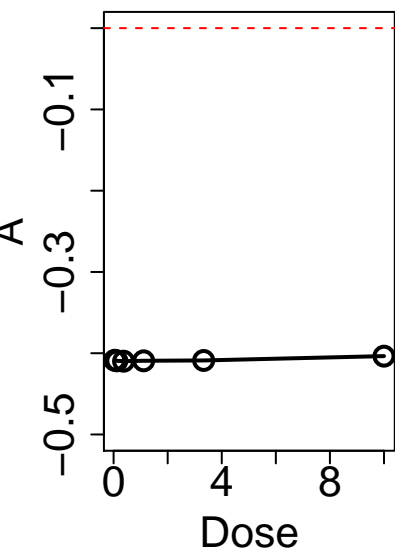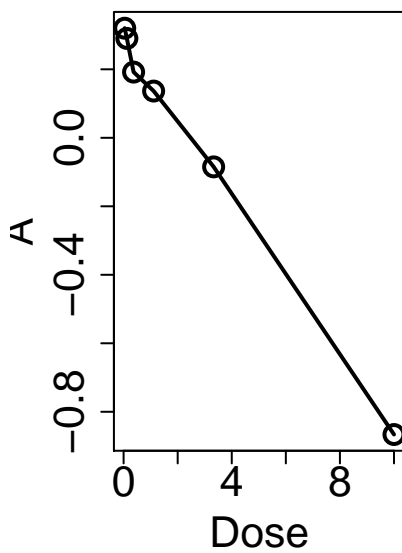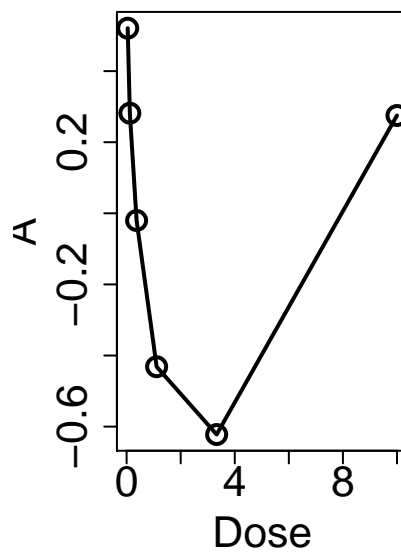

Supplement: Supplementary file 2 — Fig. S1 Second dose-dependent singular value vectors. (PDF 19 kb) [file 12859_2018_2395_MOESM2_ESM.pdf]
